# *Nodulation Signaling Pathway* 2: A Proposed Multifunctional Signaling Hub for Coordinating Plant Architecture, Rhizosphere Chemistry, and Symbiotic Associations Under Nutrient Stress

**DOI:** 10.3390/biology15191683

**Published:** 2026-09-22

**Authors:** Yanting Wang, Yanni Zhao, Ranran Liu, Shulei Wang, Yuanfeng Li

**Affiliations:** 1 College of Agriculture and Biology, Liaocheng University, Liaocheng 252000, China; liuranran0826@163.com (R.L.); wangshulei@lcu.edu.cn (S.W.); liyuanfeng@lcu.edu.cn (Y.L.); 2 Yangling Demonstration Zone Bureau of Modern Agriculture and Rural Revitalization, Yangling, Xianyang 712100, China; zhaoyanni918@163.com

**Keywords:** NSP2, nutrient stress, strigolactones, flavonoids, plant architecture, arbuscular mycorrhizal symbiosis, rhizobial symbiosis

## Abstract

Under nutrient stress, plants adjust shoot branching, root remodeling, and symbiotic associations to optimize survival and growth. The GRAS-type transcription factor NODULATION SIGNALING PATHWAY 2 (NSP2) participates in these responses through context-dependent interactions with partners: NSP1, MYB40, and SLR1, and is indirectly repressed by SMAX1 via NSP1 and SLR1. NSP2 functions differ among species: it directly binds DNA in rice (*Oryza sativa*) strigolactone (SL) biosynthesis, whereas in *M. truncatula* it acts as a co-regulator in nodulation and flavonoid regulation, with NSP1 or MYB40 providing DNA-binding activity; the NSP2–MYB module also appears conserved in barley (*Hordeum vulgare*). Through these partnerships, NSP2 is proposed to coordinate three interconnected modules influencing plant architecture, rhizosphere chemistry, and symbiotic associations. The NSP1–NSP2 heterodimer activates SL biosynthesis under phosphate deficiency, remodeling root/shoot architecture and balancing N/P uptake in rice. The NSP1–NSP2–SLR1–SMAX1 quaternary module integrates phosphate, gibberellin, and karrikin signals to orchestrate arbuscular mycorrhizal symbiosis in rice. Under nitrogen limitation and rhizobial infection, the NSP2–MYB40 module enhances flavonoid biosynthesis to promote rhizobial nodulation in *Medicago truncatula*. These findings suggest that the NSP2-centered network could be targeted to improve nutrient-use efficiency, although further field validation is required.

## Simple Summary

Plants need nutrients such as nitrogen and phosphorus to grow, but these are often in short supply in soils. To survive, plants can change their root and shoot growth, or form partnerships with beneficial soil microbes. This review focuses on a protein called NSP2, which forms different protein complexes under different nutrient conditions and helps regulate plant responses. When phosphorus is low, NSP2 works with partner proteins to adjust root and shoot growth and release chemical signals into the soil that can recruit helpful fungi. When nitrogen is scarce, NSP2 associates with a different partner to produce signals that attract nitrogen-fixing bacteria. Hormones and environmental stresses can also influence these interactions. Understanding how NSP2 functions in these context-dependent complexes may help scientists develop crops that could use nutrients more efficiently and need fewer fertilizers, which could contribute to more sustainable farming and reduced environmental pollution.

## 1. Introduction

Phosphorus (P) and nitrogen (N) are macronutrients governing plant growth and physiological metabolism [1]. P is indispensable for energy transfer, nucleic acid synthesis, photosynthesis, respiration, enzyme regulation, and signal transduction [2], whereas N provides the core constituent of proteins, nucleic acids, phospholipids, chlorophyll, and hormones [3]. In natural soils, however, both nutrients are often scarce: P readily forms insoluble precipitates, while N is lost through leaching and volatilization, and is costly to assimilate. Consequently, co-limitation by N and P is frequent in agroecosystems and represents a major constraint on global crop productivity [4].

To cope with nutrient scarcity, plants have evolved intricate sensing and signaling networks that maintain homeostasis and optimize resource allocation [5]. Two complementary adaptive strategies are deployed: internal developmental remodeling and external symbiotic recruitment [6]. Internal remodeling entails changes in plant architecture, including modification of root architecture and inhibition of shoot branching or tillering [6]. External symbiotic recruitment, particularly through symbiosis with arbuscular mycorrhizal (AM) fungi (AMF), enables plants to overcome phosphate (Pi) scarcity, as AMF facilitate Pi uptake in exchange for plant-derived carbon (C) [6]. Similarly, leguminous plants establish symbiosis with N-fixing rhizobia to secure N under limiting conditions [7].

Strigolactones (SLs), a class of carotenoid-derived phytohormones, act as a central molecular bridge linking internal developmental programs with arbuscular mycorrhizal (AM) fungi symbiosis. Originally identified as germination stimulants for parasitic weeds [8], SLs have since been recognized as multifunctional signals. Endogenously, they suppress tillering/branching and remodel root architecture [9,10]. When exuded into the rhizosphere, they promote symbiotic establishment by stimulating hyphal branching of AMF [11]. SL biosynthesis is strongly upregulated under Pi deficiency, positioning SLs as a key output of the Pi starvation response. Under Pi deficiency, SL biosynthesis is further modulated by other phytohormones—particularly gibberellin (GA) and karrikin (KAR)—which regulate SL biosynthesis and symbiotic gene expression to control AMF symbiosis establishment [12,13]. GA signaling negatively regulates SL production by promoting the degradation of DELLA protein SLR1 under Pi-limiting conditions, thereby suppressing AMF colonization in rice [13,14]. Conversely, KAR signaling, perceived through the Dwarf14-Like (D14L) receptor, acts through the transcriptional repressor SUPPRESSOR OF MORE AXILLARY GROWTH 2 1 (SMAX1) to control early AMF-responsive genes, including those involved in SL production and Myc factor recognition [13].

Flavonoids constitute another major group of rhizosphere-active secondary metabolites that mediate plant–microbe interactions. These phenylpropanoid-derived compounds are exuded by plant roots and function as signals to attract beneficial rhizobacteria, induce rhizobial nod gene expression, and modulate root microbiome composition [15]. Flavonoid biosynthesis is induced under both Pi and N limitation, suggesting that plants deploy this chemical language to recruit specific microbial partners depending on the prevailing nutrient deficiency [16]. In legumes, flavonoids serve as the first molecular signal in the symbiotic dialogue with rhizobia, being essential for successful nodulation [17].

Thus, both SLs and flavonoids function as complementary rhizosphere signals that translate internal nutrient status into external microbial communication. This raises a fundamental question: how do plants perceive nutrient limitation and coordinately regulate the biosynthesis of these two classes of signaling molecules to achieve appropriate developmental and symbiotic outcomes? A key component of this regulatory mechanism may involve a conserved transcriptional regulatory module centered on the GIBBERELLIC ACID INSENSITIVE, REPRESSOR of ga1-3, and SCARECROW (GRAS)-domain transcription factors Nodulation Signaling Pathway 1 (NSP1) and NSP2. Initially discovered as essential regulators of rhizobial nodulation in legumes [18,19,20], NSP1 and NSP2 have more recently emerged as central integrators of nutrient signals with diverse downstream responses. Among them, NSP2 is particularly notable: in legume nodulation signaling, it functions as a transcriptional co-regulator lacking intrinsic DNA-binding capacity, whereas in rice (*Oryza sativa*) SL biosynthesis, it directly binds to the promoters of SL biosynthetic genes to inducing SL production [20,21]. Despite this functional divergence, NSP2 achieves its versatility through associating with distinct transcription factors—including NSP1, MYB40, and SLR1—and through indirect regulation by SMAX1 via NSP1 and SLR1 under different nutrient and hormonal conditions. These condition-specific interactions have been shown to regulate SL biosynthesis, flavonoid production, and symbiotic gene activation [13,16,20]. However, whether these partners directly compete for NSP2, dynamically exchange within the same cell, or operate under defined prioritization thresholds remains to be experimentally determined.

In this review, we synthesize current knowledge on NSP2 as a multifaceted signaling hub that coordinates nutrient-dependent plant development and rhizosphere interactions. We first describe the structural and evolutionary basis of NSP2 function, as well as the hierarchical regulatory networks that underpin its functional plasticity. We then dissect three interconnected regulatory modules: (1) the NSP1–NSP2 heterodimer controlling SL biosynthesis, root architecture, and nutrient uptake; (2) the NSP1–NSP2–SLR1–SMAX1 quaternary module, which integrates Pi starvation, GA, and KAR signals to orchestrate AMF symbiosis; and (3) the NSP2–MYB40 module regulating flavonoid biosynthesis and beneficial microbial recruitment, including rhizobial symbiosis. Finally, we propose a conceptual framework in which NSP2, through condition-specific interactions with different partners, is hypothesized to coordinate resource allocation between growth and symbiotic engagements according to the prevailing nutrient status. We also discuss strategies for engineering this NSP2-centered regulatory network to enhance nutrient use efficiency and reduce fertilizer dependence in crops.

## 2. Structural and Functional Architecture of NSP2

### 2.1. GRAS Family and the NSP Subfamily

GRAS transcriptional regulators are a plant-specific group of proteins that play critical roles in plant development, GA signaling, and symbiotic interactions [22]. They are characterized by a highly variable N-terminal region and a conserved C-terminal domain. The C-terminal domain consists of five conserved motifs arranged in a fixed order: leucine heptad repeat I (LHRI), VHIID, leucine heptad repeat II (LHRII), PFYRE, and SAW [23]. The conserved GRAS domain consists of an α-helical cap and an α/β core subdomain, which is a shared architecture among GRAS family members [24]. The variable N-terminal sequences are involved in distinct signal transduction pathways and contribute to functional specificity [25].

NSP1 and NSP2 belong to the NSP subfamily of GRAS proteins, which is one of the 15 subfamilies [25]. Orthologs of NSP1 and NSP2 have been identified in early-diverging land plant lineages, including bryophytes such as *Marchantia* and mosses, suggesting an ancient evolutionary origin for this regulatory module [26]. However, despite this deep conservation, the DNA-binding functions of NSP1 and NSP2 exhibit remarkable divergence between legumes and rice. In legumes, NSP1 functions as a DNA-binding transcriptional activator, whereas NSP2 lacks intrinsic DNA-binding capacity [20]. Structural analyses revealed that legume NSP1 contains a conserved HCCC-type zinc finger within the PFYRE motif—a feature absent in other GRAS subfamilies—which is required for its DNA-binding activity and nodulation function [22]. NSP2 lacks this zinc finger motif and therefore cannot independently bind DNA in the legume nodulation context [22]. By contrast, in rice SL biosynthesis, it is NSP2, not NSP1, that directly binds to the promoters of SL biosynthetic genes, while NSP1 stabilizes NSP2 and enhances its transcriptional activity [21]. This functional inversion suggests that the NSP1–NSP2 module has undergone adaptive evolution across species and signaling contexts, with different partners assuming the DNA-binding role depending on the downstream targets.

The structural basis of NSP2 function has been further elucidated through recent crystallographic studies. The crystal structure of the *Medicago truncatula* NSP1–NSP2 complex at 2.4 Å resolution revealed that the two proteins assemble into a heterodimer through a small, triangular interface formed by their LHRI motifs [22]. This direct interaction is functionally significant, as NSP2 enhances the DNA-binding activity of NSP1, and disruption of this interaction compromises nodulation [22]. These findings provide a structural framework for understanding how NSP2, despite lacking DNA-binding capacity, contributes to the regulation of symbiotic gene expression in the context of legume nodulation. As discussed in Module I, the functional roles of NSP1 and NSP2 are inverted in rice SL biosynthesis, highlighting the remarkable adaptability of this regulatory module across species and signaling contexts.

### 2.2. Functional Conservation and Evolution of NSP2

The structural features of NSP2 are evolutionarily conserved across angiosperms, and this conservation underpins its diverse biological functions in both symbiotic and non-symbiotic contexts. The biological significance of these structural features was first revealed through genetic studies in legumes, in which NSP1 and NSP2 were identified as essential regulators of rhizobial nodulation, positioned downstream of calcium/calmodulin-dependent kinase (CCaMK) in the common symbiosis signaling pathway (CSSP) [18,19,25,27]. Biochemical studies revealed that they form a heterodimeric complex that binds directly to promoters of early nodulin genes such as *ENOD11* in legumes, indicating that they function as Nod factor-responsive transcriptional activators upon heterodimerization [20]. At the point downstream of CCaMK, mycorrhizal and rhizobial signaling pathways bifurcate [25]. Although NSP1 and NSP2 are not essential for AMF symbiosis, an NSP2-mediated signaling pathway promotes root colonization by AMF in *M. truncatula* [28].

NSP1 and NSP2 are not only restricted to legume plants, and their orthologs are also present in non-leguminous plants, such as rice (*Oryza sativa*), which is an arbuscular mycorrhizal host but not a rhizobial host, and *Arabidopsis thaliana*, a species that cannot establish either rhizobial or mycorrhizal symbiosis [18,19,29,30]. Moreover, legume NSP1 and NSP2 can be functionally complemented by orthologs from rice, demonstrating that the core functions of NSP1 and NSP2 are conserved across higher plants [31]. Together, these observations suggest that NSP2 fulfills conserved function in plant growth and development beyond symbiosis. This hypothesis was further confirmed by Liu et al., who demonstrated that NSP1 and NSP2 are indispensable for SL biosynthesis in both *M. truncatula* and rice, indicating that the regulation of SL biosynthesis by NSP1 and NSP2 represents an ancestral function conserved throughout angiosperm evolution [25]. Given that rice and *M. truncatula* diverged approximately 150 million years ago, this functional conservation underscores the fundamental importance of the NSP module in plant development and environmental adaptation [32]. In legumes, NSP1 and NSP2 are single-copy genes, implying that they fulfill dual regulatory functions: controlling downstream targets after rhizobium-induced signaling and regulating SL biosynthesis under non-symbiotic conditions [25]. The coordination of these distinct functions requires precise regulation at multiple levels.

### 2.3. Hierarchical Regulatory Networks Underpinning the Functional Plasticity of NSP2

NSP2 activity is controlled at multiple levels, forming a hierarchical regulatory network. At the transcriptional level, PHR transcription factors activate *NSP1* and *NSP2* expression in response to Pi starvation. At the post-transcriptional level, miR171h imposes spatial restriction on NSP2 accumulation in *M. truncatula*. Finally, at the post-translational level, NSP2 is predominantly nuclear, where context-dependent interactions with different partners influence which transcriptional program is activated.

*O. sativa* PHOSPHATE STARVATION RESPONSE (OsPHR2) functions as a master regulator of Pi homeostasis. Under Pi-sufficient conditions, *Saccharomyces cerevisiae* SYG1 and Pho81 and the mammalian Xpr1 (SPX) proteins physically interact with OsPHR2 in the cytoplasm, thereby preventing PHR proteins from entering the nucleus. Under low-Pi conditions, OsPHR2 dissociates from SPX and translocates into the nucleus, where it activates the transcription of low-Pi responsive genes [13,33]. Among the direct targets of OsPHR2 are *NSP1* and *NSP2*. Dual-luciferase reporter assays and electrophoretic mobility shift assays (EMSAs) have demonstrated that OsPHR2 directly binds to the promoters of both genes, thereby upregulating their transcription under Pi-limiting conditions [13]. This transcriptional activation provides the molecular basis for subsequent NSP1–NSP2 heterodimer formation and downstream SL biosynthesis under low Pi [13].

At the post-transcriptional layer, miR171h acts as a critical repressor shaping the spatial distribution of NSP2. Mature miR171h recognizes and cleaves NSP2 transcripts via sequence complementarity, reducing the abundance of NSP2 protein [34]. miR171h and NSP2 exhibit mutually exclusive expression patterns in root tissues: miR171h predominantly accumulates in the root elongation zone, epidermis and outer cortical layers, where it suppresses NSP2 accumulation [34]. Consequently, functional NSP2 only persists in inner cortical cells with low miR171h levels, restricting SL biosynthesis and symbiotic signaling to discrete root regions [34]. Notably, miR171h expression is tightly linked to the plant’s nutritional status: it is strongly induced under phosphate-sufficient conditions, independent of the CRE1 signaling pathway, and is further enhanced in nodulated roots under nitrogen starvation [35]. Both Myc factors and cytokinin signaling via the CRE1-MtRR1 module induce miR171h expression, establishing a negative feedback circuit to prevent excessive AM fungal colonization [34]. However, in mycorrhizal roots, NSP2 transcript levels remain elevated despite increased miR171h accumulation, and the two transcripts show a clear anti-correlation under all other tested conditions [35]. Collectively, miR171h-mediated post-transcriptional silencing imposes spatial constraints on NSP2 activity, integrating nutrient homeostasis with symbiotic regulation to safeguard the NSP2 expression domain during both arbuscular mycorrhizal and root nodule symbiosis.

Apart from transcriptional and post-transcriptional control, NSP2 activity is governed by at least two post-translational switches: nuclear translocation, which determines its access to target genes, and dynamic partner switching within the nucleus, which determines which transcriptional program it activates.

NSP2 is predominantly nuclear. Although an early study reported that NSP2-GFP expressed from a constitutive promoter localizes to the endoplasmic reticulum (ER) and nuclear envelope in the absence of symbiotic stimulation [18], subsequent evidence indicates that NSP2 is constitutively localized to the nucleus in most plant species examined, including rice and *M. truncatula* [20,21]. Once in the nucleus, NSP2 does not act alone. Depending on the biological context, NSP2 either binds DNA directly, as in rice SL biosynthesis, or functions as a transcriptional co-regulator that engages with a DNA-binding partner to determine the functional output. The identity of the DNA-binding partner may be influenced by the prevailing nutrient and hormonal context.

NSP1 is the canonical partner. NSP1–NSP2 heterodimerization is essential both for activating SL biosynthetic genes in rice under Pi deficiency and for Nod factor-induced transcriptional activation in legumes during AMF symbiosis (Table 1) [21,36]. This module thus coordinates two major outputs: it remodels plant architecture (shoot branching suppression, lateral root modulation) and promotes AMF recruitment via SL exudation under Pi deficiency, while also driving the transcriptional program required for nodule organogenesis in legumes.

MYB40 represents an alternative partner. Unlike NSP1, MYB40 is a legume-specific MYB transcription factor induced by rhizobia in the root epidermis [16]. NSP2 interacts with MYB40 during rhizobial infection to strongly upregulate flavonoid biosynthetic genes [16]. This NSP2–MYB40 module integrates symbiotic signaling with metabolic reprogramming, driving flavonoid production to promote rhizobial infection and nodulation (Table 1). Notably, this module also appears to facilitate AMF colonization under nutrient starvation, suggesting that NSP2–MYB40 may have functions beyond rhizobial symbiosis [16].

SLR1 and SMAX1 represent regulatory partners that modulate the activity of the NSP1–NSP2 complex in rice. SLR1 physically interacts with NSP2 and enhances the transcriptional activity of the NSP1–NSP2 complex, linking GA signaling to SL production (Table 1) [13]. Conversely, SMAX1 interacts with SLR1 and represses the SLR1-enhanced transcriptional activity of the NSP1–NSP2 complex (Table 1) [13]. These interactions collectively form the NSP1–NSP2–SLR1–SMAX1 quaternary module, which functions as a central regulatory hub operating downstream of Pi starvation, GA, and KAR signaling pathways to control presymbiotic transcriptional responses essential for AMF symbiosis establishment [13].

## 3. Module I: NSP1–NSP2 Heterodimer in Strigolactone Biosynthesis and Plant Architecture

It is well established that SL production in various species is significantly enhanced under Pi deficiency [37,38,39]. While the PHR transcription factors have been shown to mediate the transcriptional response to Pi starvation, the mechanism that directly links Pi sensing to SL biosynthesis remained elusive until the recent discovery of the NSP1–NSP2 heterodimer as the master transcriptional activator of SL biosynthetic genes under Pi deficiency of rice (Figure 1) [21].

### 3.1. Molecular Characterization of the NSP1–NSP2 Module

Mechanistically, in response to low-Pi stress, OsPHRs activate the transcription of NSP1 and NSP2, and the newly synthesized proteins assemble into a functional NSP1–NSP2 heterodimer [21]. Within this complex, the functional role of NSP1 and NSP2 differs from that observed in legume nodulation.

No NSP2-GFP band was detected in a co-immunoprecipitation (Co-IP) assay, suggesting that NSP2 may be degraded by the 26S proteasome in vivo [21]. Afterwards, using transgenic calli expressing Ubi:NSP1-FLAG and Ubi:NSP2-FLAG, treatment with the 26S proteasome inhibitor MG132 showed that NSP1-FLAG was detectable regardless of MG132 treatment, whereas NSP2-FLAG was only detectable after a 3-h MG132 incubation [21]. All these results indicate that NSP2 is inherently unstable in vivo.

Further dual-Luciferase reporter assays demonstrated that NSP1 alone cannot activate the DWARF 10 (D10) promoter in rice; however, upon co-expression of NSP2, NSP1 significantly boosts the transcriptional activity of the NSP1–NSP2 complex [21]. Furthermore, the NSP1–NSP2-FLAG fusion protein exhibits higher protein abundance, reduced sensitivity to MG132, and stronger transcriptional activation activity compared with NSP2-FLAG alone [21]. Moreover, this fusion protein is capable of activating multiple SL biosynthetic genes, including *D27*, *D17*, *Os900*, and *Os1400*, as confirmed by a ChIP-qPCR assay [21].

At the DNA-binding level, the functional division of labor between NSP1 and NSP2 becomes evident. EMSAs demonstrated that NSP1 alone cannot directly bind to the *D10* and *Os900* promoters in rice, whereas NSP2 and the NSP1–NSP2 fusion protein are capable of direct binding [21]. Mutations in the *NSP2*-binding motif from AATTT to CCCCC in the promoter drastically impair the transcriptional activation capacity of NSP1–NSP2, confirming that NSP2 provides the DNA-binding capacity for targeting SL biosynthetic genes [21].

Collectively, these findings demonstrate that for SL biosynthesis in rice, NSP1 stabilizes NSP2 to synergistically activate SL biosynthetic genes, with NSP2 serving as the DNA-binding subunit and NSP1 functioning as a stabilizing co-activator [21].

Notably, OsPHR2 can also bind directly to the promoters of SL biosynthetic genes and promote SL production synergistically with NSP1 and NSP2 [25,36]. This dual-layer regulation—direct activation by PHRs and transactivation via NSP1–NSP2—ensures robust SL production specifically under Pi-limiting conditions.

### 3.2. Species-Specific Conservation of the NSP–SL Regulatory Axis

The NSP–SL regulatory module is not restricted to rice. In *M. truncatula*, NSP1 and NSP2 are also required for SL biosynthesis under Pi-limiting conditions [25]. For instance, SL biosynthetic gene *D27* was downregulated in the roots of the *nsp1* mutant, and complementation with *NSP1* restored its expression [25]. Similarly, in barley, *nsp1* and *nsp2* mutants exhibit severely reduced SL production under Pi starvation, with loss of P-starvation-induced SL production [36]. Furthermore, overexpression of *MtNSP1* and *MtNSP2* in barley resulted in elevated SL levels under low-P conditions [36]. In tomato, all the known SL biosynthesis genes (*SlD27*, *SlCCD7*, *SlCCD8*, *SlMAX1*, *SlCYP722C*, and *SlCYP712G1*) were significantly upregulated in response to Pi deficiency in the wild-type, whereas this induction was markedly attenuated in *Slnsp1* and *Slnsp2* mutants, suggesting that the NSP1–NSP2 module regulates SL biosynthesis in tomato in response to Pi deficiency [40]. These findings indicate that the NSP-mediated regulation of SL biosynthesis is an ancestral function conserved across angiosperms [25].

### 3.3. SL-Mediated Shoot and Root Architectural Remodeling

The SLs produced through NSP1–NSP2-mediated biosynthesis under Pi deficiency are perceived by the receptor D14, triggering the formation of the D14–SCF^D3–D53 complex and subsequent D53 degradation via the ubiquitin-proteasome pathway [41,42,43]. This releases the repression of downstream transcription factors, leading to a coordinated suite of adaptive responses to Pi limitation.

Under Pi-deficient conditions, SL signaling inhibits shoot branching in both rice and *Arabidopsis* through conserved but distinct molecular pathways. In rice, D53 directly interacts with Ideal Plant Architecture1 (IPA1), a transcription factor that promotes tiller suppression, and inhibits its activity [44]. Upon SL perception, D53 is degraded via the proteasome, releasing IPA1. IPA1 then binds to the promoter of *OsTB1* and activates its expression, which in turn represses tiller bud outgrowth [44,45]. This reduces tiller numbers and prevents wasteful C expenditure when Pi is limiting. In *Arabidopsis*, a similar logic operates: SMXL6, 7, and 8 (the counterparts of rice D53) are degraded upon SL perception. This degradation releases transcription factors including SQUAMOSA PROMOTER BINDING PROTEIN-LIKE 9 (SPL9), SPL15, BRI1-EMS-SUPPRESSOR 1 (BES1), and TEOSINTE BRANCHED1, CYCLOIDEA, PCF4 (TCP4), which then activate *BRC1* (the *Arabidopsis* homolog of *OsTB1*) expression [46,47,48]. BRC1 subsequently induces downstream genes such as *Homeobox Protein 21* (*HB21*), *HB40*, and *HB53*, promoting ABA biosynthesis and suppressing axillary bud outgrowth [45,49,50]. Whether similar ABA-mediated downstream cascades operate in rice remains to be investigated. In both species, the core logic is conserved: SL signaling relieves repression of a transcription factor (IPA1 in rice; SPL9/15/BES1/TCP4 in *Arabidopsis*) that activates a TCP-family branching suppressor (*OsTB1*/*BRC1*) in response to Pi deficiency.

Beyond shoot architecture, SL signaling also modulates root system architecture under Pi deficiency, primarily through regulating lateral root density and root hair development. Using synthetic GR24 stereoisomers, Yuan et al. identified *CROWN ROOTLESS 1* (*CRL1*) as an early SL-responsive gene in rice roots. *CRL1* encodes a transcription factor essential for lateral root formation, and its expression is repressed by SL treatment and Pi deficiency [21]. This repression is functionally significant: *crl1* mutants no longer respond to SL treatment or Pi deficiency in terms of lateral root density, indicating that CRL1 is essential for SL-mediated lateral root development under Pi-limiting conditions [21]. SL treatment represses auxin-activated genes and auxin transporter genes, and SL-deficient mutants (*d10* and *d27*) accumulate higher IAA levels in roots [21]. This suggests that SLs modulate auxin signaling to fine-tune *CRL1* expression and lateral root development. By contrast, genetic analysis revealed that NSP1, NSP2, and D14 are not required for Pi deficiency-induced repression of crown root development, indicating that the effect of SLs on root architecture is specific to lateral roots [21]. Additionally, *OsRSL6,* an early SL-responsive gene involved in root hair development, is repressed by GR24 and Pi deficiency, suggesting a potential mechanism for SL-mediated regulation of root hair elongation under low-Pi conditions [21].

Under Pi deficiency, N absorption genes (*OsNRT*s, *OsAMT*s, and *OsNIA1*) were inhibited by Pi deficiency in the wild-type, while this repression was weakened or even abolished in the *nsp1 nsp2* mutant [21]. Conversely, *rac*-GR24 treatment induced the expression of Pi transporter genes (*OsPT*s) even in the d10 mutant [21]. Consistently, the *nsp1 nsp2* mutant accumulate less Pi than the wild-type under low-Pi conditions [21]. Together, these findings demonstrate that the NSP1–NSP2 module is essential for rebalancing N and Pi uptake under Pi deficiency, contributing to the coordination of N and Pi homeostasis when this nutrient is limiting.

Collectively, these three SL-mediated outputs—shoot suppression, root remodeling, and nutrient homeostasis—operate in concert to optimize plant fitness under Pi-limited conditions.

## 4. Module II: The NSP1–NSP2–SLR1–SMAX1 Quaternary Module in Arbuscular Mycorrhizal Symbiosis

### 4.1. SL as a Rhizosphere Signal for AMF Recruitment

As discussed in Module I, SL biosynthesis is strongly induced under Pi deficiency through the NSP1–NSP2 heterodimer in rice [21]. Beyond their endogenous roles in regulating shoot and root architecture, SLs are exuded from plant roots into the rhizosphere, where they function as key signaling molecules that mediate plant-AMF communication.

Early studies observed that the root exudates from plants grown under Pi-limiting conditions exhibited higher hyphal branching activity than those from Pi-sufficient plants, suggesting that production of this branching factor is regulated by Pi availability [51]. However, the identity of this factor remained elusive for decades due to its low concentration and instability in root exudates [11]. It was not until 2005 that the branching factor was isolated from *Lotus japonicus* root exudate and identified as the SL 5-deoxy-strigol (5DS) through spectroscopic analysis and chemical synthesis [11]. Both synthetic 5DS and GR24 induced hyphal branching in AM fungus *Gigaspora margarita* at very low concentrations, confirming that SLs act as inducers of hyphal branching in AMF [11]. This hyphal branching response is critical for establishing the physical contact necessary for subsequent fungal colonization.

The reciprocal nature of this signaling is noteworthy: plant roots exude SLs into the rhizosphere; upon perceiving plant-derived SLs, AMF synthesize and secrete their own signaling cues, namely short-chain chitooligosaccharides (COs) and lipochitooligosaccharides (LCOs). Plant roots recognize these fungal signals to trigger the conserved CSSP, which is a prerequisite for AMF colonization [28,52,53]. Nevertheless, plant species display prominent qualitative differences in their capacity to perceive COs and LCOs, determined by developmental stage and nutrient availability. Legumes such as *M. truncatula* strongly respond to both COs and LCOs, and strictly discriminate structurally distinct LCOs to separate rhizobial and AMF signals [28,36,53,54]. Previous work showed that young and nutrient-sufficient rice seedlings barely detect LCOs and only respond to COs [54,55,56,57]. Yet mature rice grown under N/Pi starvation clearly perceives LCOs, as do barley, wheat (*Triticum aestivum*), and maize [36]. In short, LCO recognition under nutrient limitation is a conserved trait of cereals, and such interspecific differences in fungal signal perception mirror distinct evolutionary strategies for AMF symbiosis.

### 4.2. GA and KAR Signaling Converge on the NSP1–NSP2 Complex via SLR1 and SMAX1

In addition to SL-mediated fungal recruitment, plant perception of AMF-derived signals and the subsequent activation of symbiosis signaling are tightly regulated by other phytohormones, particularly GA and KAR. These two phytohormone pathways converge on the NSP1–NSP2 complex through the regulatory partners, SLR1 and SMAX1, forming the NSP1–NSP2–SLR1–SMAX1 quaternary module that integrates Pi, GA, and KAR signals to control presymbiotic transcriptional responses essential for AMF symbiosis establishment in rice (Figure 1) [13].

#### 4.2.1. GA Signaling: SLR1 as a Positive Regulator of NSP Activity

Previous studies have shown that *slr1* mutants exhibit reduced root mycorrhizal colonization, implicating GA signaling in the early stages of AMF symbiosis in rice [58]. To elucidate the mechanism, Hong et al. treated rice wild-type, the SL-deficient mutant *d17*, the GA-deficient mutant *d18*, and the *d17 d18* double mutant with GA_3_ and the GR24^4DO^ (a synthetic strigolactone analog corresponding to the 4-deoxyorobanchol stereoisomer, which activates SL signaling with minimal effects on SL biosynthetic gene expression) [13]. The *d17* mutant exhibited reduced root colonization, whereas the *d18* mutant displayed colonization levels comparable to wild-type [13]. Notably, the *d17 d18* double mutant showed colonization levels similar to *d17*, indicating that GA acts upstream of SL biosynthesis in regulating AMF colonization. Consistently, expression levels of SL biosynthetic genes (*D27*, *D17*, and *Os0900*) were higher in *d18* and *d17 d18* than in wild-type and *d17* [13]. GA_3_ treatment significantly inhibited root colonization and suppressed AMF-induced expression of SL biosynthetic genes in all genotypes, whereas GR24^4DO^ treatment enhanced root colonization with minimal effects on SL gene expression [13]. These results indicate that GA negatively regulates AMF colonization, in part, through inhibition of SL production in rice.

At the molecular level, yeast two-hybrid (Y2H) and Co-IP assays revealed that SLR1 specifically interacts with NSP2, but not with NSP1 in rice [13]. Dual-Luciferase reporter assays showed that co-expression of SLR1 with the NSP1–NSP2 fusion protein enhanced the activity of rice SL biosynthetic gene reporters [13]. Conversely, GA signaling promotes SLR1 degradation via the 26S proteasome pathway, thereby reducing the transcriptional activity of the NSP1–NSP2 complex and suppressing SL biosynthesis in rice [13,14]. Consistently, expression levels of *NSP2*, GA biosynthetic gene *CPS1*, the Myc factor perception gene *MYR1*, and the SL biosynthetic gene *Os0900* were significantly higher in rice *SLR1*-overexpressing plants compared to wild-type [13]. Collectively, these findings demonstrate that GA negatively regulates SL production at the presymbiotic stage by modulating the activity of the NSP1–NSP2–SLR1 complex in rice: GA promotes SLR1 degradation, thereby reducing NSP1–NSP2 transcriptional activity, while SLR1 itself positively regulates this process by interacting with NSP2 and enhancing complex activity.

#### 4.2.2. KAR Signaling: SMAX1 as a Repressor of the NSP Complex

Mutations in the KAR receptor D14L abolish both AMF colonization and transcriptional responses to AMF, indicating that D14L is indispensable for AMF perception in rice [59]. A similar symbiosis-defective phenotype was observed in the *d3* mutant (the rice ortholog of *MAX2*) [59,60]. Subsequent genetic analyses revealed that SMAX1, a downstream repressor of the KAR signaling pathway, negatively regulates AM symbiosis in rice: *smax1* mutants exhibit enhanced fungal colonization and elevated expression of AM-induced genes even in the absence of AMF inoculation, and the colonization defects of *d14l* and *d3* mutants are suppressed in *smax1 d14l* and *smax1 d3* double mutants [61]. Notably, a set of evolutionarily conserved genes present in AM-host species is dependent on functional SMAX1 [9]. Collectively, these findings indicate that the removal of the SMAX1 suppressor is required for transcriptional activation of the ancient AM symbiotic program in rice.

The mechanistic link between KAR signaling and the NSP1–NSP2 complex has been established through multiple lines of evidence. Knockout of either *NSP1* or *NSP2* abolishes AMF-induced upregulation of KAR-signaling-regulated early response genes (KERGs) (including *Os0900*, *ZAS1*, *MYR1*, and *D14L2*) in rice, whereas most other AMF-induced early response genes (OERGs) remain intact, indicating that the NSP1–NSP2 complex is specifically required for KAR-dependent transcriptional activation of KERGs [13]. At the protein level, pull-down and Co-IP assays confirmed that SMAX1 physically interacts with NSP1 in rice [13]. Importantly, further analysis via Y2H, pull-down, and Co-IP assays revealed that SMAX1 also interacts with SLR1 [13]. Moreover, a dual-luciferase reporter assay revealed that this interaction allows SMAX1 to repress the SLR1-enhanced transcriptional activity of the NSP1–NSP2 complex [13]. Genetic epistasis analysis revealed that NSP1 and NSP2 function downstream of SMAX1 in rice: the enhanced AMF colonization and KERG expression observed in *smax1* mutants are abolished when *nsp1* or *nsp2* mutations are introduced [13]. Overexpression of *NSP2* partially bypasses SMAX1-mediated repression, but cannot fully compensate for the loss of D14L function, suggesting that additional KAR signaling outputs beyond SMAX1 degradation are required for full AMF colonization in rice [13].

#### 4.2.3. Integration of Pi, GA, and KAR Signals by the Quaternary Module

The interactions among NSP1, NSP2, SLR1, and SMAX1 collectively form the NSP1–NSP2–SLR1–SMAX1 quaternary module. This module integrates three major signaling inputs: Pi starvation (via PHR-mediated transcriptional upregulation of NSP1 and NSP2), GA signaling (via SLR1 stability and its interaction with NSP2), and KAR signaling (via SMAX1 degradation and its repression of the NSP complex) (Figure 1) [13]. Through this integration, the module ensures that AMF symbiosis is initiated under Pi-limiting conditions, when GA signaling is attenuated (stabilizing SLR1) and AMF-triggered KAR signaling relieves SMAX1-mediated repression. However, while KAR signaling-mediated SMAX1 degradation is necessary for triggering presymbiotic transcriptional responses, it alone is not sufficient for successful colonization; activation of the CSSP by AMF-derived LCOs is also required.

## 5. Module III: The NSP2–MYB Module in Flavonoid Biosynthesis and Rhizobial Symbiosis

### 5.1. Flavonoids as the First Molecular Signal in Rhizobial Symbiosis

Flavonoids are best known for their function in the legume–rhizobial symbiosis, where they act as the initial molecular signal that initiates symbiotic communication [17]. Under N-limiting conditions, legume roots release specific flavonoids—including flavones, chalcones, and isoflavonoids—into the rhizosphere [16]. Compatible rhizobia perceive these compounds, which activate bacterial nod genes and triggers the production of LCO Nod factors [15]. Plant LysM receptor-like kinases recognize Nod factors and initiate a signaling cascade that includes calcium spiking in root epidermal cells, activation of the CSSP, and downstream responses such as root hair curling, infection thread formation, and nodule organogenesis [7].

Flavonoids also contribute to host specificity in the legume–rhizobial symbiosis. Different legume species produce distinct flavonoid exudate profiles, and many rhizobia carry multiple NodD homologs with varying flavonoid-recognition specificities. This specificity ensures that only compatible rhizobial strains are triggered to synthesize Nod factors, preventing the plant from committing resources to unproductive symbiotic interactions [15,17]. Chalcones and flavones secreted by the legume root epidermis are particularly effective in inducing Nod-factor production, which then triggers symbiotic calcium spiking and downstream transcriptional reprogramming in plant roots [15].

Beyond their role in initiating symbiosis, flavonoids also influence nodule development. Silencing of *chalcone synthase* (*CHS*) in *M. truncatula*, the first committed enzyme in flavonoid biosynthesis, nearly abolishes nodulation, demonstrating that flavonoid production is essential for successful symbiosis [17]. Additionally, *chalcone-O-methyltransferase* (*ChOMT*) is preferentially expressed in root hairs and promotes nodulation in *M. truncatula* [16]. Flavonoids also regulate auxin transport in a spatially controlled manner, facilitating cortical cell division and nodule organogenesis [17].

Despite the established importance of flavonoids in the legume–rhizobia symbiosis, the transcriptional mechanisms that control flavonoid biosynthesis during nodulation have remained largely unclear until the recent identification of the NSP2–MYB40 module [16].

### 5.2. NSP2–MYB40 Module: Transcriptional Regulation of Flavonoid Biosynthesis

Transcriptional regulation of flavonoid biosynthesis is well-studied in non-leguminous model species such as *Arabidopsis* [62], where MYB transcription factors function as central regulators of the phenylpropanoid pathway [63,64,65,66]. However, whether similar regulatory logic applies to legume nodulation—where flavonoids serve as symbiotic signals rather than stress-induced metabolites—remains unclear [67,68].

NSP1 and NSP2 are established regulators of both rhizobial and mycorrhizal symbioses [7,18,19,20], initially identified for their role in activating early nodulation genes, including the master regulator NIN [19,69]. In the AM symbiosis, they regulate SL biosynthesis [21,25]. However, SLs are dispensable for nodulation [70,71], suggesting that NSP1 and NSP2 must control additional metabolites during rhizobial symbiosis. Transcriptomic data pointed to a possible link between GRAS proteins and flavonoid production [72], prompting a direct test of whether NSP1 and NSP2 directly regulate flavonoid biosynthesis during nodulation in *M. truncatula*.

Gao et al. found that many flavonoid biosynthetic genes—*phenylalanine ammonia lyase* (*PAL*), *chalcone synthase* (*CHS*), *chalcone O-methyltransferase* (*ChOMT*), and *isoflavone malonyltransferase* (*IMaT*)—are induced under nutrient starvation and rhizobial inoculation, but this induction is partially or completely suppressed or lost in *nsp1* and *nsp2* mutants in *M. truncatula* [16]. Metabolite measurements confirmed the phenotype: isoliquiritigenin and 4,4′-dihydroxy-2′-methoxychalcone (DHMC) were significantly reduced in *M. truncatula nsp* mutant roots. Transactivation assays further revealed that NSP2, not NSP1, activates the promoters of these flavonoid genes *M. truncatula*, positioning NSP2 as the principal activator [16].

The DNA-binding partner for NSP2 was identified in *M. truncatula* through promoter analysis, which uncovered a conserved cis-element matching a MYB-binding motif [16]. Among the MYB genes induced by nutrient starvation and rhizobial nodulation, MYB40—a legume-specific MYB factor—emerged as the candidate [16]. Its expression pattern parallels that of flavonoid biosynthetic genes, with strong induction in root hairs and cortical cells upon rhizobial inoculation [16]. EMSAs confirmed direct binding of MYB40 to *CHS1* and *ChOMT1* promoters [16].

Physical interaction between NSP2 and MYB40 during rhizobial infection was supported by multiple assays in *M. truncatula*: Y2H, split luciferase complementation, and Co-IP all confirmed that NSP2 (but not NSP1) interacts with MYB40 [16]. Both proteins colocalize to the nucleus, and structural modeling suggested that the VHIID, PFYRE, and SAW domains of NSP2 form a groove that accommodates the C-terminal α-helix of MYB40 [16].

Functionally, NSP2 enhances MYB40-mediated transcriptional activation (Figure 1). ChIP-qPCR showed that MYB40 enriches the *ChOMT1* promoter, and co-expression of NSP2 increases this enrichment [16]. Mutating the MYB40 binding sites in the *ChOMT1* promoter abolished rhizobial-induced reporter activity, confirming that MYB40 binding is essential for activation [16].

Genetic evidence supports the physiological relevance of this module. *M. truncatula myb40* mutants showed reduced infection threads and fewer nodules under low rhizobial density, along with decreased isoliquiritigenin and DHMC levels [16]. Exogenous flavonoid application rescued the nodulation defect, confirming that it results from reduced flavonoid production [16]. Conversely, co-expression of MYB40 with a miR171h-resistant version of NSP2 (miRR-NSP2) enhanced nodulation under suboptimal rhizobial conditions [16].

Collectively, these findings establish the NSP2–MYB40 module as a transcriptional regulator that links symbiotic signaling to flavonoid biosynthesis (Figure 1), directing flavonoid production to the sites and stages where rhizobial infection and nodulation require it.

### 5.3. The NSP2–MYB Module in AMF Symbiosis and Its Evolutionary Implications

The role of the NSP2–MYB module is not confined to rhizobial symbiosis. Because nodule symbiosis evolved from the more ancient AMF association and flavonoids operate in both interactions [73,74,75], the possibility that the NSP2–MYB40 module contributes to AMF symbiosis was investigated. A potential complication is that NSP1 and NSP2 regulate SL biosynthesis so any mycorrhizal phenotype in myb40 mutants might in principle arise from altered SL production. However, no change in D27 or CCD8 expression was detected in myb40 mutants of *M. truncatula*, making it less likely that the mycorrhizal phenotype arises primarily from altered SL production, although other aspects of SL biosynthesis, metabolism, exudation, or signaling cannot be fully excluded [16]. When inoculated with *Rhizophagus irregularis*, *M. truncatula* myb40 mutants showed roughly 65% less root colonization than wild-type plants, yet arbuscule morphology remained normal [16]. Thus, MYB40 appears to act during early stages of symbiotic signaling rather than in arbuscule development.

Additional evidence from barley supports evolutionary conservation of the module. Loss of *HvNSP1* or *HvNSP2* abolished nutrient deficiency-induced expression of *HvPAL* and *HvCHS*, whereas overexpression of *M. truncatula NSP1* or *NSP2* in barley enhanced their expression [16]. Importantly, *HvNSP2* complemented the *M. truncatula nsp2-2* mutant in nodulation assays, and HvNSP2 interacted physically with HvMYB7 (a MYB40 homolog in barley) in *Nicotiana benthamiana* [16]. The presence of an NSP2–MYB module in barley strongly suggests that this regulatory logic is conserved across angiosperms.

These observations point to an evolutionary scenario in which the NSP2–MYB module originated in the context of the ancestral AMF symbiosis and was subsequently co-opted in legumes to regulate the N-fixing symbiosis with rhizobia [16]. This co-option provided legumes with a mechanism to fine-tune N acquisition in response to nutrient availability, while the ancestral function in AMF symbiosis persists in both legumes and cereals.

## 6. A Proposed Model for NSP2-Mediated Integration of Environmental Signals and Coordination of Adaptive Outputs: Conserved and Species-Specific Features

The three regulatory modules described above—SL biosynthesis and plant architecture remodeling (Module I), AMF symbiosis (Module II), and flavonoid biosynthesis and rhizobial symbiosis (Module III)—are not independent pathways operating in isolation. Rather, we propose that they are coordinated through a common regulatory hub centered on NSP2 (Figure 1). This section synthesizes these modules into a working hypothesis, proposing that NSP2 may function as a hierarchical decision-making hub that integrates multiple environmental signals—particularly Pi and N availability, as well as the presence of AMF—to coordinate resource allocation between growth and symbiotic engagements. We emphasize that this framework is a conceptual model based on independent interaction studies; direct competition among these partners, dynamic exchange within the same cell, and quantitative prioritization thresholds remain to be experimentally demonstrated. In addition to these mechanistic uncertainties, the strength of evidence also varies across species and modules. Module I (NSP1–NSP2–SL) is primarily based on genetic and biochemical evidence from rice [21], with conserved roles reported in *M. truncatula* [25], barley [36], and tomato [40]. Module II (NSP1–NSP2–SLR1–SMAX1) is also primarily characterized in rice for the quaternary module [13], whereas the downstream events—LCO perception, calcium oscillation, and symbiotic gene activation—are supported mainly by evidence from barley and *M. truncatula* [36]. Module III (NSP2–MYB40) is based primarily on *M. truncatula*, with conserved evidence from barley [16]. Importantly, NSP2 functions, DNA-binding capacities, and symbiotic competences differ among species. The extent to which this network is conserved across all species therefore remains to be determined.

### 6.1. Environmental Inputs: Phosphate, Nitrogen, and AMF Presence

We hypothesize that NSP2 integrates at least three major classes of environmental signals that collectively determine the plant’s adaptive strategy.

Pi status serves as the primary gatekeeper. Under Pi deficiency, OsPHR2 activates the transcription of *NSP1* and *NSP2* [21]. The newly synthesized proteins assemble into a functional NSP1–NSP2 heterodimer in rice, which directly binds to the promoters of SL biosynthetic genes, leading to a dramatic increase in SL production [21]. This Pi-dependent transcriptional activation provides a molecular basis for both root architectural remodeling (Module I) and AMF recruitment (Module II) (Figure 1). Conversely, under Pi-sufficient conditions, *NSP1* and *NSP2* expression remains low, and both SL biosynthesis and AMF symbiosis are suppressed.

N status modulates the partner association of NSP2. Under N-limiting conditions, flavonoid biosynthesis is induced in *M. truncatula* [16]. During rhizobial infection in legumes, NSP2 interacts with MYB40 to enhance the activation of flavonoid biosynthetic genes, driving flavonoid production to promote rhizobial infection and nodulation (Module III) (Figure 1) [16]. Importantly, flavonoid biosynthesis is induced under both Pi and N limitation, suggesting that plants deploy this chemical language to recruit specific microbial partners depending on the prevailing nutrient deficiency [16].

AMF presence modulates the NSP1–NSP2 complex through KAR signaling. AMF inoculation activates the KAR signaling pathway of rice via the D14L receptor, leading to SMAX1 degradation and relieving SMAX1-mediated repression of the NSP1–NSP2–SLR1 complex [13]. Importantly, the transcriptional responses triggered by KAR signaling are strictly dependent on the NSP1–NSP2 complex, positioning NSP2 as the essential downstream executor of AMF-induced transcriptional reprogramming [13].

Beyond N and P: the emerging role of C availability. Symbioses are energy-intensive since AMF symbiosis consumes photosynthetic products, and rhizobial symbiosis imposes similar C costs on the host plant. Strikingly, the expression of NSP1 responds to changes in light quality during nodulation of soybean (*Glycine max*) [76], and NSP2 expression exhibits circadian rhythmicity in *M. truncatula* [77]. These observations suggest that NSP1 and NSP2 may regulate symbiosis not only as a function of N/P availability, but also as a function of C availability—determined by photosynthetic capacity [78]. This adds an additional regulatory layer: even when N or P is limiting, symbiosis may only proceed when sufficient C is available to sustain the microbial partners. Thus, we propose that NSP1/NSP2 may function as gatekeepers of symbiosis, only allowing engagement with symbiotic microorganisms when nutrients are limiting and when photosynthetic capacity is sufficient to sustain the increased C demand [78]. This C-related regulatory mechanism represents a relatively understudied but critical dimension of NSP2 function.

Additional environmental cues: salt stress and iron status. Emerging evidence indicates that environmental factors beyond nutrient availability also influence NSP function. Salt stress and iron levels modulate symbiotic N fixation through post-translational modification of NSP1 in soybean [79,80]. These findings suggest that NSP1 and NSP2 integrate a broader range of environmental signals—including abiotic stresses—to fine-tune symbiotic outcomes. Thus, we hypothesize that NSP2 functions not merely as a nutrient sensor but as a multi-signal integrator that evaluates the plant’s overall physiological and environmental status.

### 6.2. The Integration Hub: NSP2 as a Central Processor

We propose that the functional versatility of NSP2 may lie in its ability to associate with different partners in response to distinct environmental signals, thereby routing information toward different downstream outputs.

Under Pi deficiency, NSP2 partners with NSP1 to form the NSP1–NSP2 heterodimer in rice, directly activating SL biosynthetic genes and triggering both plant architectural remodeling and AMF recruitment. The PHR2–NSP1/2–SL axis thus represents the core Pi-responsive pathway that coordinates internal developmental adjustments with external symbiotic signaling.

Under N limitation in legumes, and particularly during rhizobial infection, NSP2 interacts with MYB40 to activate flavonoid biosynthetic genes and promote rhizobial infection and nodulation. Notably, this module also facilitates AMF colonization under nutrient starvation, suggesting that NSP2–MYB40 may have functions beyond rhizobial symbiosis.

SLR1 and SMAX1 may function as regulatory modulators of the NSP1–NSP2 complex activity in rice. SLR1 interacts with NSP2 and enhances NSP1–NSP2 activity, linking GA signaling to SL production. SMAX1, in contrast, represses NSP1–NSP2 and NSP1–NSP2–SLR1 activity. AMF-triggered KAR signaling relieves this repression by promoting SMAX1 degradation. Thus, we propose that SLR1 and SMAX1 may function as a hormonal rheostat that fine-tunes NSP1–NSP2 activity in response to GA and KAR signals.

We hypothesize that this partner association is not a default process but may be governed by the prevailing nutrient and hormonal environment. NSP1 may be preferentially involved under Pi-limiting conditions, whereas N limitation together with rhizobial infection may promote NSP2–MYB40 interaction. AMF presence, through SMAX1 degradation, may further modulate this balance. Such context-dependent associations could allow NSP2—a single protein—to coordinate a range of outputs that are not always aligned [13,16,21]. However, direct evidence for partner switching and prioritization is still lacking, and this proposal requires further experimental validation.

### 6.3. Decision Outputs: From Signal Integration to Adaptive Responses

Through context-dependent partner associations, we propose that NSP2 may translate integrated environmental signals into a coordinated set of adaptive outputs.

When Pi deficiency is the predominant signal in rice, the PHR–NSP1/2–SL axis is activated, leading to suppression of shoot branching, root architecture remodeling, and enhanced SL exudation for AMF recruitment [21]. This suite of responses constitutes a conservative exploration strategy: the plant restricts above-ground C expenditure while simultaneously investing in root system development and symbiotic preparation. This strategy thus positions the plant to maximize Pi acquisition through developmental plasticity and microbial engagement.

When Pi deficiency coincides with AMF presence, KAR signaling is activated, triggering SMAX1 degradation and subsequent derepression of the NSP1–NSP2–SLR1 complex [13]. This results in the expression of KERGs, including genes involved in SL production and Myc factor recognition [13]. However, SMAX1 degradation alone is not sufficient for successful colonization; activation of the CSSP by AMF-derived LCOs is also essential [13]. This two-step requirement—KAR signaling for transcriptional priming and CSSP activation for colonization—ensures that AMF symbiosis proceeds only when both the plant is transcriptionally competent and the fungus is physically present. This represents a symbiotic engagement strategy, initiated only when internal nutrient status and external fungal signals are both permissive.

Under N deficiency and rhizobial infection, the NSP2–MYB40 module is implicated in promoting flavonoid biosynthesis for nodulation in *M. truncatula* [16]. This targeted recruitment strategy is more specialized than AMF engagement: it requires precise flavonoid profiles for compatible rhizobia and thus involves greater molecular specificity and resource investment per interaction.

When both Pi and N are limiting, the plant must allocate limited resources between AMF symbiosis and rhizobial symbiosis. We propose that NSP2 may be positioned at the nexus. The relative intensities of Pi and N deficiency signals—transduced through PHR-dependent transcriptional activation and MYB40 partner selection, respectively—may influence which pathway predominates [16,21]. The energetic costs of maintaining both symbioses further constrain this choice [78]. Consistent with the proposed role of NSP1 and NSP2 as ‘nutrient-responsive regulators’ that integrate environmental signals and nutrient availability to modulate symbiosis [78], NSP2 appears to function as a key decision node in resource allocation between competing symbiotic programs.

C availability imposes an additional regulatory layer [78]. Even under N/P limitation, symbiosis may proceed only when photosynthetic capacity can sustain the increased C demand [78]. This suggests that NSP1 and NSP2 may act as gatekeepers of symbiosis: engagement with microbial partners is permitted only when nutrients are limiting and C supply is sufficient [78]. This C-related regulatory layer remains relatively unexplored but represents a critical dimension of NSP2 function.

Together, these observations support a proposed “basal setpoint and modulatory override” model of NSP2-mediated coordination. Pi status sets the basal setpoint: when Pi is limiting, the plant defaults to a conservative exploration strategy. AMF presence provides a modulatory override: KAR signaling commits the plant to AMF symbiosis. N status modulates partner association: NSP2 associates with MYB40 under N limitation. C availability acts as a permissive condition, determining whether the plant has sufficient energy to engage in symbiosis at all. Through this proposed hierarchical integration, NSP2 may enable plants to balance growth, stress tolerance, and symbiotic engagements according to the prevailing environmental conditions. However, direct competition among these partners, dynamic exchange within the same cell, and quantitative prioritization thresholds remain to be experimentally demonstrated.

## 7. Conclusions, Implications, and Future Perspectives

### 7.1. Conclusions

NSP2 functions as a central signaling hub that integrates nutrient status, hormonal signals, and symbiotic cues to coordinate plant architecture, rhizosphere chemistry, and microbial interactions. Through context-dependent partner associations—engaging NSP1 under Pi deficiency in rice, MYB40 under N limitation in *M. truncatula* and rhizobial infection in rice, and SLR1 in response to GA signaling, while being indirectly modulated by SMAX1 via NSP1 and SLR1—NSP2 is proposed to translate environmental information into distinct developmental and symbiotic outputs. The three interconnected modules described in this review—SL biosynthesis and plant architecture (Module I), AMF symbiosis (Module II), and flavonoid biosynthesis and rhizobial symbiosis (Module III)—collectively illustrate how a single transcriptional regulator can orchestrate multiple adaptive strategies through context-dependent protein–protein interactions. This mechanistic framework positions NSP2 as a proposed molecular processor that may evaluate the plant’s physiological status and environmental context to coordinate resource allocation between growth and symbiosis.

### 7.2. Potential Agricultural Implications

The mechanistic insights into NSP2 function provide several promising strategies for sustainable crop improvement. The NSP1/2–SL module coordinates N and P uptake under Pi deficiency by repressing N transporter genes while activating Pi transporter genes in rice [21]. These findings suggest that modulating NSP activity might help optimize N–P nutritional balance in crops, although this remains to be tested under field conditions. The NSP1–NSP2–SLR1–SMAX1 quaternary module may provide strategies for enhancing AMF symbiosis under Pi-sufficient conditions [13]. Loss of SMAX1 function or overexpression of NSP2 in rice enhances AMF colonization with minimal negative effects on plant growth in laboratory studies, suggesting that these genetic manipulations could help decouple AMF symbiosis from Pi status—a key bottleneck for leveraging mycorrhizal associations in fertilized agricultural soils [13]. However, whether this can be translated to crops and field conditions requires further investigation. Overexpression of MtNSP2 in barley partially relieves P-mediated suppression of AMF colonization, which may contribute to improved nutrient capture efficiency and reduced fertilizer loss, but field validation is needed [36]. The NSP2-MYB40 module links symbiotic signaling to flavonoid biosynthesis in *M. truncatula*, providing a molecular framework that could inform future crop engineering, though practical applications remain to be demonstrated [16]. Understanding this ‘nutritional checkpoint’ mechanism offers theoretical targets for designing next-generation crops with potentially reduced fertilizer dependence, pending further mechanistic and field validation.

### 7.3. Limitations, Controversies, and Future Directions

Despite the integrative framework proposed in this review, several key aspects remain unresolved or controversial. First, direct competition among NSP2 partners, dynamic exchange within the same cell, and quantitative prioritization thresholds have not been experimentally demonstrated in any species. The model therefore remains a working hypothesis rather than an established mechanism. Second, SMAX1 is an indirect regulator that interacts with NSP1 and SLR1, not with NSP2 directly; its placement within the NSP2-centered network should be interpreted accordingly. Third, N deficiency alone has not been shown to directly switch NSP2 from NSP1 to MYB40; current evidence supports a role for NSP2–MYB40 during rhizobial infection under N-limiting conditions. Alternative models should also be considered: NSP2 may not function as a single decision-making hub, but rather as a shared component of independent complexes operating in different cell types or developmental stages.

Several key questions include: (1) How are different NSP2 complexes assembled dynamically in response to combinatorial signals? What determines the threshold at which one signal overrides another? Addressing this will require single-cell proteomics, in vivo competition assays, and quantitative interaction dynamics to test whether NSP2 truly switches partners, prioritizes outputs, and operates through defined thresholds. (2) Do additional NSP2 partners exist that mediate responses to other environmental cues, such as salt stress or iron deficiency? (3) What is the precise mechanism by which C availability is integrated into the NSP regulatory network? Addressing these questions will require integrated approaches combining single-cell transcriptomics, structural biology, protein interactomics, and field-based multi-omics analyses.

Ultimately, NSP2-mediated nutrient management strategies, if validated in crops, could help regulate symbiosis and nutrient acquisition, contributing to more sustainable agriculture. However, substantial further research is needed to translate these mechanistic findings into practical applications.

## Figures and Tables

**Figure 1:**
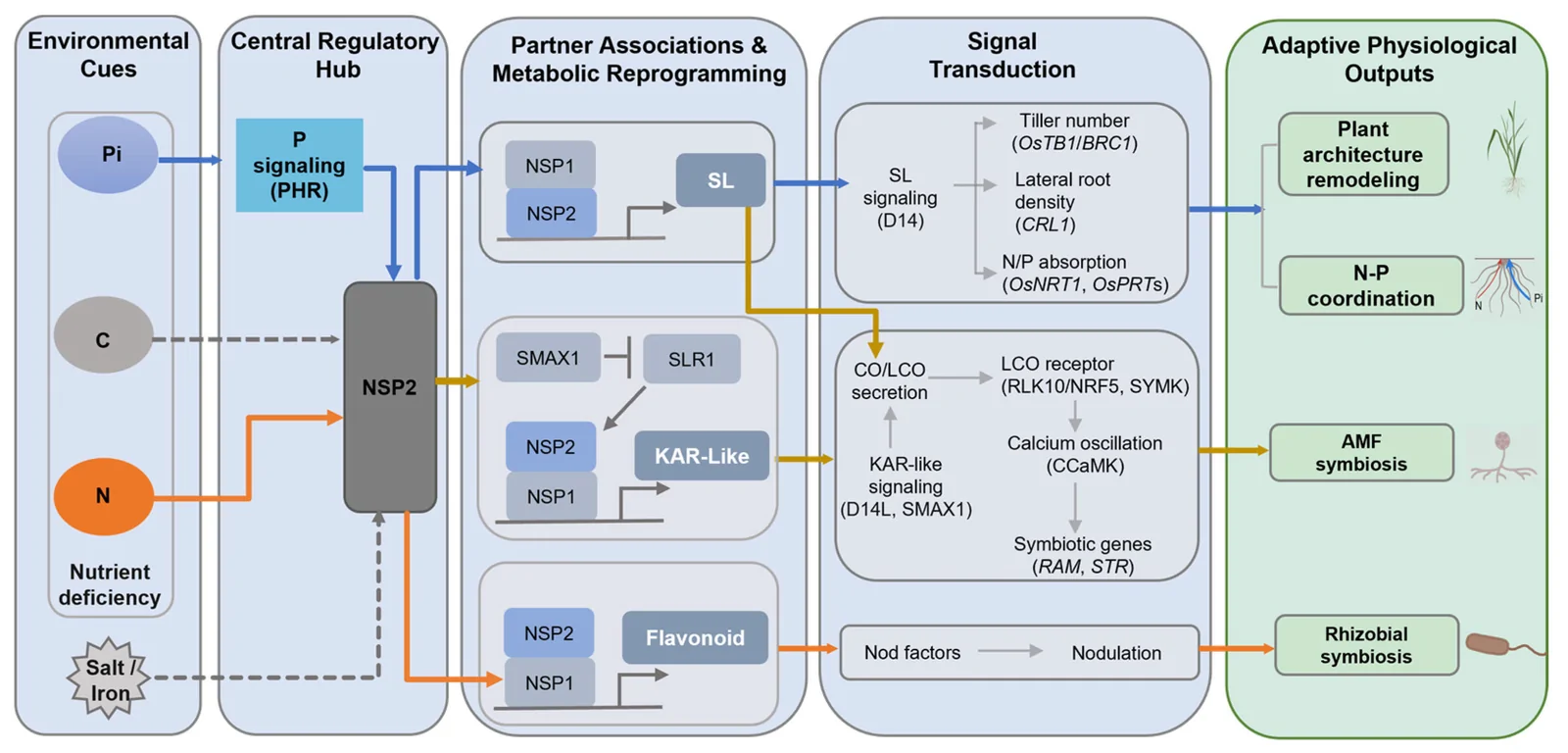
An evidence-based working model for NSP2-mediated integration of nutrient, hormonal, and symbiotic signals. NSP2 acts as a signaling hub that coordinates plant architecture remodeling, arbuscular mycorrhizal fungi (AMF) symbiosis, and rhizobial symbiosis. Under Pi deficiency, NSP2 associates with NSP1 to activate strigolactone (SL) biosynthesis (primarily in rice). Under N limitation and rhizobial infection, NSP2 engages MYB40 to drive flavonoid biosynthesis for rhizobial nodulation (in *Medicago truncatula*). Central to AMF symbiosis is the NSP1–NSP2–SLR1–SMAX1 quaternary module (primarily in rice): SLR1 enhances NSP1–NSP2 activity, while SMAX1 represses it; AMF-triggered KAR signaling via D14L degrades SMAX1, relieving repression and activating KERGs. Downstream lipochitooligosaccharide (LCO) perception, calcium oscillation (*CCaMK*), and symbiotic gene activation is supported mainly by barley (*Hordeum vulgare*) and *M. truncatula*. Even when N or P is limiting, symbiosis may proceed only when photosynthetic capacity can sustain the increased carbon demand. Additionally, salt stress and iron status may also modulate symbiotic outcomes through post-translational modification of NSP1. Solid arrows indicate activation; T-bars indicate repression, and dashed lines indicate proposed or less-characterized connections.

**Table 1:** Species-specific functions, interacting proteins, and downstream outputs of NSP2 in nutrient-responsive developmental and symbiotic pathways.

Regulatory Hub	Interacting Protein	Regulatory Signal	Species	Effect on NSP2 Complex	Biological Process	Module
NSP2	NSP1	Pi deficiency	*Oryza sativa* (primary; conserved in *Medicago truncatula*)	NSP1 stabilizes NSP2 and enhances its activity	SL biosynthesis → Plant architecture remodeling, AMF recruitment	I
NSP2	SLR1	GA signaling (low GA → SLR1 accumulation)	*Oryza sativa*	SLR1 enhances NSP1–NSP2 transcriptional activity	SL biosynthesis → AMF symbiosis promotion	II
NSP2	NSP1 and SLR1	KAR signaling (AMF → SMAX1 degradation)	*Oryza sativa* (KAR–SMAX1 module)*; Hordeum vulgare and Medicago truncatula* (downstream LCO perception and symbiotic genes)	SMAX1 (via NSP1/SLR1) represses NSP1–NSP2–SLR1 activity; AMF promotes SMAX1 degradation, relieving repression	KERGs → AMF colonization	II
NSP2	MYB40	N limitation + rhizobial infection	*Medicago truncatula* (conserved in *Hordeum vulgare*)	NSP2–MYB40 interaction enhances flavonoid gene activation during rhizobial infection	Flavonoid biosynthesis → Rhizobial nodulation	III

## Data Availability

No new data were created or analyzed in this study. Data sharing is not applicable to this article.
